# Reliability of the fMRI-based assessment of self-evaluation in individuals with internet gaming disorder

**DOI:** 10.1007/s00406-021-01307-2

**Published:** 2021-07-17

**Authors:** Patrick Bach, Holger Hill, Iris Reinhard, Theresa Gädeke, Falk Kiefer, Tagrid Leménager

**Affiliations:** 1 Department of Addictive Behavior and Addiction Medicine, Central Institute of Mental Health, Medical Faculty Mannheim/Heidelberg University, Mannheim, Germany; 2 Feuerlein Center on Translational Addiction Medicine (FCTS), University of Heidelberg, Heidelberg, Germany; 3 Institute of Sports and Sports Science, Karlsruhe Institute of Technology, Karlsruhe, Germany; 4 Department of Biostatistics, Central Institute of Mental Health, Medical Faculty Mannheim/Heidelberg University, Mannheim, Germany

**Keywords:** Self-evaluation, Reliability, Intraclass correlation, fMRI, Dice, Jaccard

## Abstract

The self-concept—defined as the cognitive representation of beliefs about oneself—determines how individuals view themselves, others, and their actions. A negative self-concept can drive gaming use and internet gaming disorder (IGD). The assessment of the neural correlates of self-evaluation gained popularity to assess the self-concept in individuals with IGD. This attempt, however, seems to critically depend on the reliability of the investigated task-fMRI brain activation. As first study to date, we assessed test–retest reliability of an fMRI self-evaluation task. Test–retest reliability of neural brain activation between two separate fMRI sessions (approximately 12 months apart) was investigated in *N* = 29 healthy participants and *N* = 11 individuals with pathological internet gaming. We computed reliability estimates for the different task contrasts (self, a familiar, and an unknown person) and the contrast (self > familiar and unknown person). Data indicated good test–retest reliability of brain activation, captured by the “self”, “familiar person”, and “unknown person” contrasts, in a large network of brain regions in the whole sample (*N* = 40) and when considering both experimental groups separately. In contrast to that, only a small set of brain regions showed moderate to good reliability, when investigating the contrasts (“self > familiar and unknown person”). The lower reliability of the contrast can be attributed to the fact that the constituting contrast conditions were highly correlated. Future research on self-evaluation should be cautioned by the findings of substantial local reliability differences across the brain and employ methods to overcome these limitations.

**Supplementary Information:**

The online version contains supplementary material available at 10.1007/s00406-021-01307-2.

## Introduction

Converging lines of evidence implicate deficits in an individual’s self-concept as a relevant factor in the development of internet gaming disorders (IGD). Due to their social and concomitantly anonymous characteristics, reports portray internet games as a tempting pastime to compensate a negative self-concept. The self-concept can be regarded as a relatively stable cognitive representation (i.e., knowledge system and beliefs) about the own person [25, 29]. It is defined as the subjective evaluation of one’s own physical appearance (physical self-concept); social competences (social self-concept); the capability to recognize, express, and regulate one’s feelings (emotional self-concept); and skills for reaching academic goals (academic self-concept) [21]. Mummendey (2006) assumes that the self-concept evolves from comparisons between the subjective view of oneself and the ideal self (i.e., how one would like to be). The ideal self is mainly influenced by an individual’s environment, such as family members, peer groups, society, and media. A negative self-concept—defined as a high discrepancy in the evaluation between the subjective self and ideal self—is often reported to be associated with gaming and other internet related disorders [16, 18, 31, 32]. A recent review on self-esteem and self-concept in gaming disorders, reported stable associations between gaming disorders and negative physical and academic self-concept domains [17]. Functional imaging studies have tried to identify the neural correlates of the self-concept by applying self-referential and self-recognition paradigms. During self-referential tasks, participants are asked to evaluate their personality traits, physical appearance, preferences, or thoughts. Resulting neural activation patterns are compared to those that emerge during the evaluation of one’s ideal self, a close friend, a famous person, or a foreign person [7, 13, 19]. The neural activations during self-referential paradigms are regarded as a functional network underlying different self-concept aspects (e.g., reflecting on one’s own social competencies can be regarded as part of the social self-concept). Self-recognition paradigms, in which participants see pictures of their own face or body relative to faces or bodies of others [22], involve unconscious comparisons between one’s own physical appearance and that of others. It can be assumed that these comparisons mirror neurobiological correlates of the physical self-concept.

A meta-analysis of Hu et al. [10] compared neural correlates of self-face recognition and self-referential paradigms in healthy participants to identify distinct and common neural regions underlying self-referential and self-recognition. Processing one’s own face relative to the face of another person, induced activation in the right inferior frontal gyrus (IFG); the bilateral fusiform gyrus; the inferior temporal gyrus; the bilateral insula; the right postcentral and supramarginal gyrus (SMG); the anterior cingulate (ACC); and the right superior occipital and angular gyrus. The meta-analysis also indicated that, across studies, self-referential paradigms induced brain activation in the bilateral ACC; the middle frontal gyrus and the superior temporal gyrus; the precuneus as well as the left inferior parietal gyrus. The conjunction analysis of both tasks revealed shared activation in the right ACC and in the left insula and IFG [10]. The results of the meta-analysis also demonstrated that self-referential and self-recognition tasks induce activation in regions of the temporoparietal junction (TPJ), extending over several cortical areas, including posterior portions of the superior temporal gyrus and adjacent parietal regions in the supramarginal and angular gyri [10]. Studies in addicted gamers revealed increased activation in the right inferior parietal lobule [14] and a decrease in the right inferior frontal gyrus [3] during self-reflection. Choi et al. (2018) assessed the self-concept in addicted adolescent gamers compared to healthy controls during self-referential tasks. The authors observed a decrease in the inferior frontal gyrus in the addicted group, indicating that addicted gamers might find it more difficult to retrieve information regarding their self-concept. Furthermore, Kim et al. (2018) found an increase in activation in the inferior parietal lobule in the addicted group during self vs. ideal self-reflection. The authors interpreted their findings as an increased identification with the real self, as compared to the ideal self, in individuals with gaming disorders. The investigation of self-concept-related characteristics via self-evaluation tasks seems to be a promising approach to further elucidate the neurobiological basis of gaming disorders; however, currently there are no data on the reliability of fMRI tasks that assess self-concept in individuals with IGD. Reliability of an fMRI task, however, is an important prerequisite for capturing individual neural correlates of the self-concept and for establishing associations between neural processes and behavior. Furthermore, it is used to predict future behavior in gaming disorders, based on neural activation patterns. Elliot and colleagues (2019) conducted a meta-analysis of fMRI studies and pointed out that the overall reliability of fMRI tasks across different task categories, designs, and study groups was low [4]. A study by Infantolino et al. (2018) also indicated that low reliability of fMRI tasks might result when fMRI task contrasts are computed by subtracting two correlated task conditions, even when the constituting task conditions show excellent reliability. This is because much of the shared “true” variance is removed when subtracting two task conditions from one another, while the error variance is summed [11, 24]. Hence, we set out to assess the reliability of neural responses during a video-based fMRI paradigm assessing neural correlates of physical, social, and emotional aspects of the self-concept in young adults. The video paradigm combines self-referential and self-recognition aspects. During the fMRI session, video clips of the participant, a close friend, and an unknown person are presented. The protagonists in the videos introduce themselves and talk about topics related to the self-concept, such as their positive personality traits (emotional and social self-concept); their expectations of others (social self-concept); as well as their future goals (academic self-concept). We assume that the comparison of neural activation between the self and other conditions mirrors neural correlates of the self-concept. To our knowledge this is the first paradigm measuring self-concept-related aspects by combining self-referential and self-recognition paradigms. We tested this paradigm in the framework of a one-year longitudinal study in a sample of healthy participants as well as individuals with pathological internet game use.

## Methods

### Study sample and patient subgroups

A total of *N* = 40 male individuals (*n* = 11 pathological [problematic and addicted] gamers and *n* = 29 controls) were included in the current analyses. Initially, *N* = 83 participants enrolled in the study and completed baseline assessment. Of those, *N* = 40 returned for a second assessment after 12 months. *N* = 40 participants provided complete datasets. Participants were recruited between March 2016 and June 2019 (trial registration: DRKS 00009439). All procedures were carried out in accordance with the Declaration of Helsinki. The local ethics committee (application number 2014-602 N-MA) approved the study procedures and all participants provided informed written consent. Individuals were recruited via advertisement and outpatient care for pathological gamers in the Central Institute of Mental Health, Mannheim, Germany. Between the first (T1) and second assessment (T2), participants did not receive any specific intervention. The average time span between T1 and T2 was 396 days (SD = 67). Abstinence from substance use was monitored through drug urine screening at each assessment.

Participants were required to be aged between 18 and 27 years and had to be right-handed. Pathological gamers were excluded if they met any of the following exclusion criteria: (i) comorbid axis I disorders in the preceding year aside from nicotine-dependence and IGD, assessed using the Structured Clinical Interview for DSM-IV Axis I Disorders (SCID) [33] and the Assessment of Internet and Computer Game Addiction (AICA) [34]; (ii) treatment with psychotropic or anticonvulsive medications; (iii) severe neurological or physiological disease (such as, but not limited to stroke, aneurysm, dementia, epilepsy, liver cirrhosis); (iv) negative urine drug test on the day of assessment; or (v) contraindications for MRI scans (i.e., pace-makers, metal implants, tattoos).

### Assessment

Participants underwent two assessment sessions, both including psychometric measures and fMRI. All participants completed questionnaires on (and after) the assessment day, including the Rosenberg Self-Esteem Scale [27], the Scale for Social Anxiety and Social Competence Deficits (SASKO; [15]), the Emotional Competence Questionnaire [26], and the Empathy Quotient [1]. Diagnosis of internet gaming addiction as well as problematic usage was evaluated with the Assessment of Internet and Computer game Addiction-Checklist (AICA-C > 13 for addictive usage and AICA-S > 6 and < 13 for problematic usage; [34]). After the first assessment, participants underwent interviews and filled out questionnaires every three months. After 12 months, participants were assessed via fMRI once again. Before the second scan, the exclusion criteria were reconfirmed. Participants were excluded if they had developed a comorbid axis I disorder (other than nicotine-dependence and IGD, in the preceding year); if they underwent treatment with psychotropic or anticonvulsive medications; or if they had suffered severe neurological or physiological disease in the preceding 12 months.

### fMRI self-evaluation task

The paradigm comprised video clips of the participant themselves, an age-matched familiar person, and an unknown person. The task was programmed with the software Presentation Version 16.3 (Neurobehavioral Systems, Albany, Calif., USA). During a video session, participants and their close friend were asked to introduce themselves and talk about different topics related to their person. Four videos of each condition (self, a familiar, and an unknown person) comprised the following topics: (1) personal introduction (instruction: “Introduce yourself: name, age, family, etc.”); (2) positive character traits (instruction: “What are your personal strengths and hobbies?”); (3) personal values and expectations of other people (instruction: “What is important to you concerning your fellow humans?”); and (4) future goals (instruction: “Where do you see yourself in five years from now, what did you achieve?”). The videos, with duration of 15 s each, were recorded in advance with a Panasonic high-definition video camera (Type HC-V707) and converted using VSDS Free Video Editor software (Version 3).

**Fig. 1:**
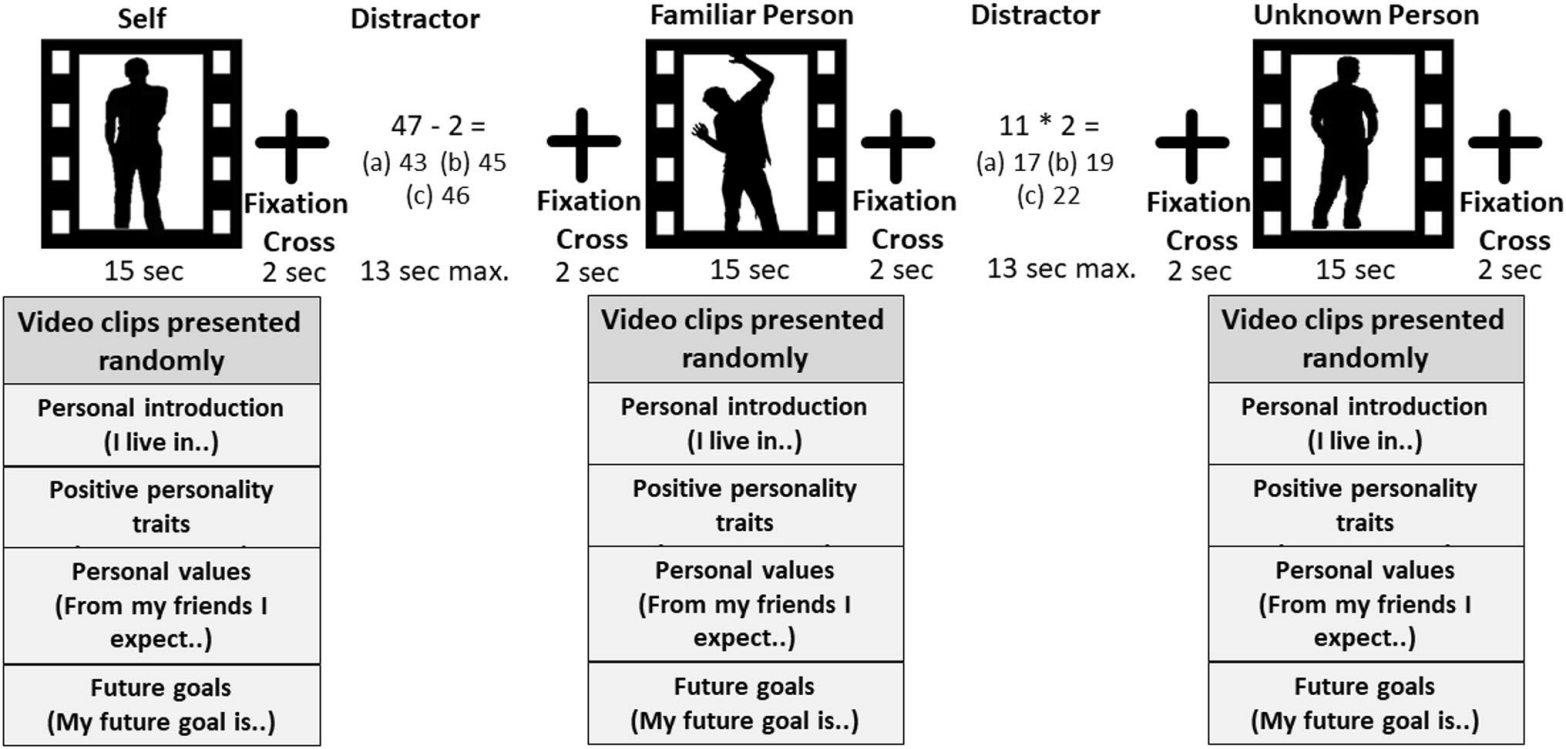
Depiction of the self-evaluation block-design paradigm

The fMRI paradigm was conducted in a block design. Each paradigm block consisted of a video clip regarding one topic from one specific condition. All blocks were presented in a randomized order. Every participant watched 12 video clips in total (three conditions comprising four videos each). Each video clip was followed by a fixation cross (two seconds) and a distractor (calculation task with a maximum duration of 13 s), where participants had to move the cursor to select an answer. Then, another fixation cross appeared before the subsequent video clip began. The distractor was used to create distance between the previous videos’ content. The total paradigm took a minimum of four and a maximum of 8 min, depending on how fast the participants solved the calculation task (see Fig. 1).

### MRI acquisition

MRI data acquisition was performed on a 3.0 Tesla MR scanner (SIEMENS MAGNETOM Trio) with a standard multi-channel receiver head coil (12-channel). During the functional self-concept MRI task, 205 volumes were acquired by applying a T2*-weighted echo-planar imaging (EPI) sequence [repetition time (TR) = 2410 ms, echo time (TE) = 25 ms, flip angle (FA) = 80°, field of view (FOV) = 192 mm × 192 mm, matrix size 64 × 64, 42 slices, slice thickness = 2.00 mm, distance factor = 50%, and voxel size = 3 × 3 × 2 mm]. Three-dimensional T1-weighted structural images (Magnetization Prepared Rapid Acquisition Gradient Echo, MPRAGE) were collected over 8 min. The T1-weighted anatomical scans comprised 192 sagittal slices (flip angle: 9˚; repetition time: 2.3 ms; echo time: 3.03 ms; field of view 256 × 256; voxel size, 1 mm × 1 mm × 1 mm). The automated Siemens Multi-Angle Projection (MAP) Shim corrected magnetic field inhomogeneity. Presentation software (Version 16.3, Neurobehavioral Systems, Inc., Albany, CA, USA) was used for both the registration of scanner triggers and the recording of behavioral responses. All participants viewed the video clips through a tilted mirror placed above their heads. During the assessment, the test persons wore foam ear plugs and headphones. Prior to the assessment, participants underwent a hearing test to adjust the sound of the video clips if necessary. After completion of the scan, participants were asked to rate the sound quality of the videos on a scale from 0 to 10. One patient, who rated the sound quality under 7, was excluded from the analyses.

### fMRI pre-processing and statistical analyses

The functional images were pre-processed according to standard procedures implemented in the statistical parametric mapping software for Matlab (SPM, Wellcome Department of Cognitive Neurology, London, UK) version 12. The first five scans of every measurement were discarded to avoid artifacts due to magnetic saturation. We conducted slice time correction, followed by spatial realignment and unwarping. A phase map correction was applied to correct geometric distortions, using a voxel displacement map that was computed from a gray field mapping sequence using the VDM utility in SPM12. Movement correction was conducted using standard SPM12 parameters and images were normalized to the standard tissue probability template provided in SPM12. Smoothing was conducted using an isotropic Gaussian kernel for group analysis (8 mm Full Width at Half Maximum). The following procedures were carried out to assess the quality of pre-processed functional MRI data. Motion correction and realignment parameters, as well as results from the normalization procedure, were assessed by two independent trained members of the study team. Datasets of participants were excluded if the spatial realignment or movement correction parameters indicated excessive motion (> 3 degrees of rotation or > 3 mm movement in any axis) or if visual inspection indicated poor fitting to the standard TPM template. The first-level statistics were computed for each participant, modelling the different experimental conditions: (i) self, (ii) familiar person, (iii) unknown person, and (iv) distractor task in a generalized linear model including six motion parameters as covariates. The general view of the self-concept is that of a stable cognitive representation (i.e., knowledge system and beliefs) about one’s subjective self in comparison to an ideal self, the latter of which is formed by the environment. In line with this view, the neural correlates of the self-concept were operationalized by subtracting brain activation during the presentation of videos of oneself from the brain activation during the presentation of videos of familiar and unknown persons (i.e., self > familiar person and unknown person). Thus, apart from the contrast images for (i) self vs. implicit baseline; (ii) familiar person vs. implicit baseline; (iii) unknown person vs. implicit baseline; (iv) distractor condition vs. implicit baseline; and (v) self vs. familiar and unknown person. The contrast between self and familiar person + unknown person was computed using the contrast weights (2 -1 -1 0) .

Previous studies suggested that difference measures suffer from low inherent reliability when the constituting conditions are correlated [11]. Hence, we also estimated reliability separately for the “self”, “familiar other”, and “unknown other” contrast conditions.

#### Analyses of self-concept-related measures

We tested the stability of self-concept measures (i.e., with SASKO, the Emotional Competence Questionnaire, the Rosenberg Self-Esteem Scale, and the Empathy Quotient) by assessing differences between the first and second experimental session (*t* tests for dependent samples) for pathological (problematic and addicted) gamers and healthy controls separately. Furthermore, we assessed test–retest reliability of self-concept measures by computing the intraclass correlation coefficient between the first and second session.

#### Analyses of group-level fMRI activation

On a group level, imaging data were analyzed using full factorial models with the factor time (first and second scan) to assess the congruence of task effects on the group-level brain activation over time. This was accomplished by determining brain areas that show higher brain activation in response to viewing videos of the own person compared to brain activation when viewing videos of familiar and foreign persons (contrast: “self > familiar and unknown person”). In addition, group-level brain activation patterns were analyzed for the constituting task conditions separately (i.e., responses to videos of the “self”); a familiar person (contrast: “familiar person”); and an unknown person (contrast: “unknown person”) at each time point. We applied a whole-brain family-wise error rate correction of *p*_FWE_ < 0.05 at the cluster level to correct for multiple comparisons.

#### Reliability measures

To assess longitudinal test–retest reliability of the self-evaluation fMRI task, we computed global and local measures of reliability. All reliability analyses were conducted using the fmreli toolbox for SPM12 [8]. Individual contrast images of the different task conditions served as input for the reliability analyses. Dice and Jaccard coefficients were analyzed within the framework of an ANOVA with the contrast condition set as four-level within-subject factor (i). self; (ii). familiar person; (iii). unknown person; (iv). self > familiar + unknown person and the experimental group set as two-level between-subject factor [(i). healthy individuals; (ii). IGD].

#### Intraclass correlation coefficient

Voxel-wise reliability of each contrast condition was estimated by computing the intraclass correlation coefficient (ICC) between the first and second assessment points. The ICC tests whether the magnitude of brain activation in each voxel is stable between the first and the second fMRI scan. Fleiss (1986) proposed that ICCs lower than 0.4 indicate poor reliability; ICCs between 0.4 and 0.6 indicate fair reliability; ICCs between 0.6 and 0.75 indicate good reliability; and ICCs with values higher than 0.75 indicate well to excellent reliability [6]. The ICC sets within-subject variance (σ^2^_within_) in relation to between-subject variance (σ^2^_between_). The ICC(3,1)-type was proposed as being the most appropriate for assessing single site longitudinal fMRI datasets [23]. Hence, we used the ICC(3,1)-type [28], defined as:

$$ICC = \frac{{\left( {\sigma^{2}_{{{\text{between}}}} - \sigma^{2}_{{{\text{within}}}} } \right)}}{{\left( {\sigma^{2}_{{{\text{between}}}} + \sigma^{2}_{{{\text{within}}}} } \right)}}.$$

ICC values were computed for the contrasts of “self”, “familiar person”, “unknown person”, and the contrasts “self > familiar and unknown person”. We computed ICCs for every brain voxel and generated thresholded ICC brain maps to identify brain areas that show good (ICC > 0.6) and good to excellent (ICC > 0.75) reliability. Furthermore, we computed additional atlas-based mean ICC values for a standard set of anatomical brain regions (see below).

#### Similarity

Similarities in the fMRI activation maps from the first and second scans were determined. The analysis captures the resemblance of two brain activation maps based on the alignment of high vs. low brain activation values across the brain. The authors of the fmreli toolbox propose that this method could be used to quantify within-subject and between-subject similarities of brain activation without requiring an a priori (and potentially arbitrary) statistical threshold. A high within-subject similarity supports the notion that individuals can be re-identified based on their neural brain activation patterns. The resulting coefficients are correlation coefficients that range from a “perfect” negative relationship (− 1.00) to a “perfect” positive relationship (1.00). In the past, studies have suggested that subjects can be successfully identified based on their neural activation pattern if the within-subject similarity exceeds all between-subject association coefficients of the same participant [5, 8]. The similarity analyses, therefore, complement the computation of the ICC, which allow inferences on a group level, providing additional information on the stability and resemblance of brain activation at an individual participant level.

#### Pearson’s correlation

We computed the mean voxel-wise Pearson’s correlation coefficients between the “self”, “familiar other”, and “unknown other” contrast conditions using the procedures provided in the fmreli toolbox. This step was taken to assess the correlation between the different task condition contrasts. This is important due to the fact that the reliability of a contrast between two conditions is limited in the case of high correlation between the activation patterns of the constituting contrast conditions.

#### Jaccard and dice coefficients

The modified Jaccard coefficient is a commonly used measure in fMRI reliability studies. It can be interpreted as the percentage of overlapping significant voxels above a predefined threshold (e.g., *p* < 0.001) within all significant voxels. The Jaccard coefficient is defined as the ratio of intersection between the number of three-dimensional image voxels, which were found to be activated in the first fMRI assessment (A) and the replication (B), divided by the size of the union of the voxel sets of A and B [12, 20].

$${\text{Jaccard}}\left( {A, B} \right) = \frac{{\left| {A \cap B} \right|}}{{\left| {A \cup B} \right|}} = \frac{{\left| {A \cap B} \right|}}{{\left| A \right| + \left| B \right| - \left| {A \cap B} \right|}}.$$

Another measure of global reliability or overlap between super-threshold voxels is the Dice coefficient. It is calculated as the number of super-threshold voxels that overlap between sessions A and B (see above) divided by the average number of significant voxels across sessions A and B (see above):

$${\text{Dice}}\left( {A, B} \right) = \frac{{2\left| {A \cap B} \right|}}{\left| A \right| + \left| B \right|}.$$

Both, Jaccard and Dice coefficients range from no overlap (0) to perfect overlap (1) between super-threshold voxels; however, currently there is no consensus on specific values or cut-offs that would differentiate between “poor” and “good” values [2]. In accordance with previous studies, the current analyses used a threshold of *p* < 0.001. Jaccard and Dice coefficients were determined for every patient by comparing the baseline and the second fMRI results for the different contrast images. Resulting values were exported into the IBM SPSS statistics software (version 25.0) and effects of contrast conditions were tested using a repeated measures analysis of variance model with contrast condition as within-subject factor.

#### Atlas- and ROI-based summary measures

To facilitate the assessment of local differences in reliability, we computed the mean ICC for *N* = 116 anatomical regions, specified in the automatic anatomic labeling (AAL) atlas [30]. ICC values were extracted from the ROIs using the data extraction routine of the MarsBar software package (http://marsbar.sourceforge.net/); then, these data were exported into the IBM SPSS statistics software (version 25.0) for further analyses.

## Results

### Reliability of psychometric self-concept-related measures

Demographic, gaming, and self-concept-related psychometric data for both time points of *N* = 40 participants are shown in Table 1. The mean period between the two fMRI scans was 396 days (SD = 67). As expected, pathological gamers showed significantly higher AICA_30 and AICA_lifetime scores than healthy controls at T1 and T2. The AICA_30 score showed a significant decrease from T1 to T2 for the pathological gaming group but not for the healthy control group. Regarding between-group differences, pathological gamers rated their social anxiety of feeling rejected higher, compared to the control groups in T2. Both subgroups did not differ in other self-concept-related measures. Furthermore, the majority of self-concept-related measures did not show a significant change over time, which indicates stability (see Table 1). Only the subscale assessing emotional regulation (EKF-RE) showed a significant increase from T1 to T2 in pathological gamers. The stability of self-concept-related measures was further supported by high test–retest reliability estimates (see Table 2).

**Table 1:** Sample description

	T1					T2					T1–T2			
	Total score	Pathological gamers (*n* = 11)	Healthy controls (*n* = 29)	*t* value	*p* value	Total score	Pathological gamers (*n* = 11)	Healthy controls (*n* = 29)	*t *value	*p* value	Pathological users *t* value	*p* value	Healthy controls *t* value	*p* value
Age (SD)	21.23 (2.51)	21.45 (3.05)	21.14 (2.33)	− 0.353	0.726	22.34 (2.12)	22.72 (2.94)	22.19 (2.37)	− 0.597	0.554	− 9.037	< 0.001*	− 13.25	< 0.001*
AICA_30 (SD)	5.15 (5.89)	13.09 (5.55)	2.14 (1.79)	− 0.641	0.007*	2.65 (2.94)	4.63 (3.59)	190 (230)	− 2.865	0.007*	4.087	0.002*	0.462	0.647
AICA_lifetime (SD)	13.70 (8.36)	22.27 (5.73)	10.45 (6.77)	− 5.125	< 0.001*	13.65 (9.33)	23.19 (6.19)	10.03 (7.64)	− 5,094	< 0.001*	− 0.396	0.700	00.405	0.688
Years of education (SD)	13.92 (1.90)	14.09 (1.92)	13.86 (1.92)	− 0.336	0.739	–	–	–	–	–	–	–	–	–

Self-concept-related measures														
SASKO: speaking (SD)	8.40 (5.92)	10.64 (6.91)	7.55 (5.38)	− 1.33	0.202	7.78 (5.04)	9.63 (5.85)	6.86 (4.56)	− 1.589	0.120	.840	0.421	1.204	0.238
SASKO: rejection (SD)	7.95 (5.27)	10.36 (5.83)	7.03 (4.83)	− 1.839	0.074	6.81 (4.71)	9.45 (6.15)	6.04 (3.71)	− 2.152	0.038*	0.897	0.391	1.528	0.138
SASKO: interaction (SD)	6.10 (4.75)	7.36 (7.00)	5.62 (3.62)	− 1.03	0.307	5.50 (3.58)	5.91 (3.78)	5.41 (3.50)	− 0.377	0.711	1.168	0.270	0.328	0.745
SASKO: information (SD)	6.08 (3.82)	8.36 (4.80)	5.20 (3.04)	− 2.032	0.063	6.18 (3.91)	7.82 (4.40)	5.66 (3.60)	− 1.456	0.119	0.482	0.640	− 0.982	0.334
SASKO: loneliness (SD)	2.15 (2.50)	2.73 (2.76)	1.93 (2.40)	− 0.898	0.375	1.84 (2.42)	2.36 (2.11)	1.72 (2.47)	− 0.814	0.454	0.510	0.563	1.063	0.297
EKF-EE (SD)	56.90 (8.27)	56.73 (9.81)	56.97 (10.18)	0.067	0.947	58.05 (9.21)	57.36 (11.78)	58.33 (8.19)	0.291	0.773	− 0.398	0.699	− 0.400	0.692
EKF-EA (SD)	62.552 (8.27)	61.18 (10.57)	63.03 (7.38)	0.628	0.534	62.06 (14.27)	58.32 (22.32)	63.59 (7.21)	0.757	0.464	0.467	0.651	0.029	0.977
EKF-RE (SD)	48.25 (6.89)	47.18 (8.11)	48.66 (6.48)	0.599	0.553	51.08 (6.94)	53.09 (7.57)	50.26 (6.64)	− 1.146	0.259	− 2.444	0.035*	− 1.090	0.285
EKF-EX (SD)	51.25 (11.18)	50.73 (13.88)	51.44 (10.25)	0.180	0.858	51.98 (13.92)	51.12 (20.29)	52.33 (10.88)	0.240	0.812	− 0.100	0.922	− 0.410	0.685
Rosenberg self-worth (SD)	22.90 (4.87)	21.36 (6.76)	23.48 (3.93)	1.238	0.223	23.76 (4.76)	23.09 (3.70)	24.04 (4.01)	0.551	0.585	− 2.000	0.074	− 0.583	0.565
Empathy (SD)	36.13 (8.03)	37.45 (9.85)	35.62 (7.36)	− 0.640	0.526	36.14 (9.75)	39.10 (9.99)	35.00 (9.61)	0.803	0.265	− 0.425	0.681	0.782	0.442

Differences in demographic, gaming, as well as self-concept-related characteristics between pathological (problematic and addicted) gamers and healthy controls at both time points (*t* tests for independent samples) and between the time points (*t* tests for dependent samples)

*AICA_30* severity of computer game addiction during the last 30 days, *AICA_Lifetime* lifetime usage of computer games, *SD* standard deviation, *SASKO* Social Anxiety and Social Competence Deficits, *EKF-EE* recognizing and understating own emotions, *EKF-EA* recognizing and understanding others’ emotions, *EKF-RE* regulation and control of own emotions, *EKF-EX* emotional expressiveness; *t*  two-sample *t* test statistics, ﻿* = *p* < 0.05

**Table 2:** Intraclass correlation coefficients of self-concept-related measures

	Total sample *N* = 40		Pathological gamers (*n* = 11)		Healthy controls (*n* = 29)	
	Intraclass correlation coefficient	*p* value	Intraclass correlation coefficient	*p* value	Intraclass correlation coefficient	*p* value
SASKO: speaking	0.820	< 0.001﻿*	0.810	0.001*	0.809	< 0.001﻿*
SASKO: rejection	0.762	< 0.001﻿*	0.843	< 0.001﻿*	0.665	< 0.001﻿*
SASKO: interaction	0.631	< 0.001﻿*	0.730	0.003﻿*	0.545	0.001﻿*
SASKO: information	0.727	< 0.001﻿*	0.668	0.009﻿*	0.728	< 0.001﻿*
SASKO: loneliness	0.845	< 0.001﻿*	0.664	0.009﻿*	0.908	< 0.001﻿*
EKF-EE	0.552	< 0.001﻿*	0.881	< 0.001﻿*	0.390	0.017﻿*
EKF-EA	0.481	0.001﻿*	0.319	0.156	0.717	< 0.001﻿*
EKF-RE	0.461	0.001﻿*	0.478	0.058	0.502	0.002﻿*
EKF-EX	0.709	< 0.001﻿*	0.712	0.005﻿*	0.705	< 0.001﻿*
Rosenberg self-worth	0.787	< 0.001﻿*	0.905	< 0.001﻿*	0.679	< 0.001﻿*
Empathy	0.464	0.002﻿*	0.475	0.070	0.453	0.009﻿*

*SASKO* Social Anxiety and Social Competence Deficits, *EKF-EE* recognizing and understating own emotions, *EKF-EA* recognizing and understanding others’ emotions, *EKF-RE* regulation and control of own emotions, *EKF-EX* emotional expressiveness, ﻿* ﻿= *p* < 0.05

### Group-level brain activation

Analyses of brain activation across both groups (*N* = 40) indicated significant self-concept associated brain activation (contrast: “self > familiar and unknown person”) in the bilateral insula; the anterior and medial cingulum; the IFG; the operculum; the bilateral putamen; the claustrum; the superior motor area; the precentral gyrus and the STG; the right globus pallidus; the superior and medial frontal gyri; the supramarginal gyrus; the postcentral gyrus; and the inferior parietal lobe (see supplementary Table S1). The patterns of brain activation were replicated, when analyzing both groups separately (see supplementary Table S1). Between-group comparisons did not reveal significant differences in brain activations at T1 or T2. Longitudinal comparison of the brain activation during first and second fMRI did not show a significant change in brain activation over time [two-tailed: increase over time (T1 < T2) or decrease (T1 > T2); see supplementary Table S1]. Detailed results of the group-level analyses for the constituting task conditions (self, familiar person, and unknown person) are depicted in the supplementary Figure S1. In short, self-related activation patterns were detected in both groups in the insula as well as in the superior and inferior temporal gyrus. For the familiar and unknown condition, activation was recorded in the middle and superior temporal gyrus.

### Reliability analyses

#### Reliability of the contrast “self > familiar and unknown person”

**Fig. 2:**
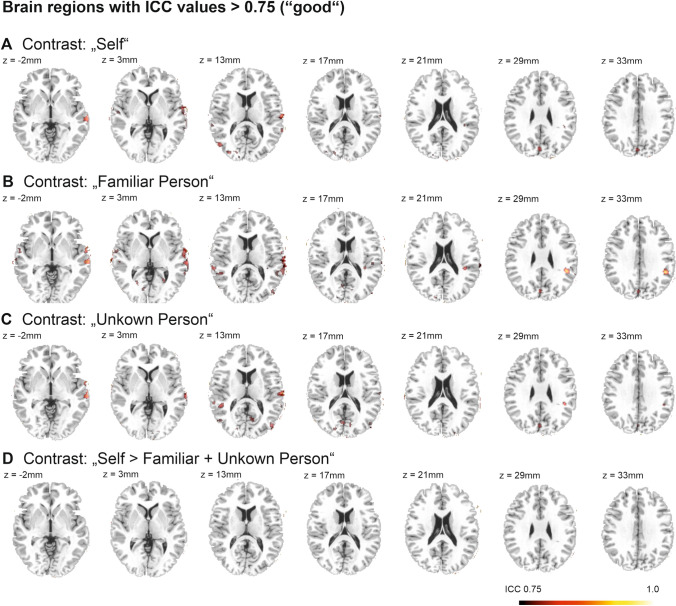
Depiction of brain areas that show good to excellent reliability for the different task contrasts: (**A**) “Self”, (**B**) “Familiar Person”, (**C**) “Unknown Person” and (**D**) “Self > Familiar + Unknown Person” [Intraclass correlation coefficient (ICC) > 0.75], when performing pooled analyses of the whole dataset of *N* = 40 participants

**Fig. 3:**
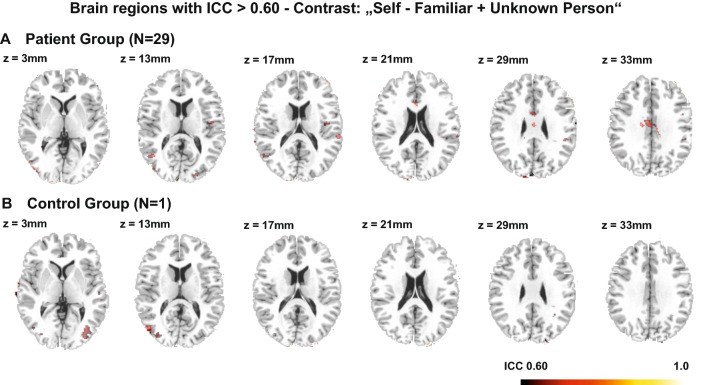
Depiction of brain areas that show moderate to good reliability (0.75 > ICC > 0.60) for the contrast “self > familiar and unknown person” in (**A**) the patient group and (**B**) the control group

For the pooled study sample (*N* = 40) and in both study groups separately, the mean ICC values of the contrast “self > familiar and unknown person” were under the threshold of moderate reliability (ICC < 0.4, see Table 3). This finding, however, came as no surprise as we had assumed that brain activation in areas, which are unrelated to the fMRI task, and the construct of self-evaluation could not be replicated in its magnitude. This resulted in a low overall ICC value across the whole brain. Both groups did not significantly differ in mean ICC values. Thresholded ICC maps illustrated that the local reliability of the contrast “self > familiar and unknown person” did not surpass the threshold for good reliability, neither in the pooled sample, nor when considering both experimental groups separately (ICC > 0.75, see Fig. 2 and supplementary Figures S2 and S3; ICC maps are available at https://identifiers.org/neurovault.collection:9777). However, several brain areas showed moderate to good reliability (0.75 > ICC > 0.60, see Fig. 3). In the patient group these areas included the right middle and anterior cingulum; the right superior temporal gyrus, including parts of the TPJ; the bilateral middle and inferior temporal gyrus; the left fusiform gyrus; the left insula; and the right inferior occipital gyrus. In the control group the areas included the bilateral middle occipital gyri as well as the right superior and middle temporal gyrus, including parts of the TPJ.

**Table 3:** Mean ICC values for different contrast conditions for the pooled sample and both study groups separately

		Contrasts			
		self	Familiar person	Unknown person	Self > familiar + unknown person
Pooled group (*N* = 40)	Mean ICC (SD)	0.24 (0.24)	0.30 (0.24)	0.25 (0.24)	0.06 (0.21)
Controls (*N* = 29)	Mean ICC (SD)	0.25 (0.24)	0.28 (0.24)	0.27 (.24)	0.07 (0.21)
Patients (*N* = 11)	Mean ICC (SD)	0.25 (0.23)	0.24 (0.22)	0.21 (0.21)	− 0.03 (0.18)

The atlas-based summary of mean ICC values for 120 brain regions for the contrast “self > familiar and unknown person” (collapsed across both groups) showed that no brain region surpassed an average mean ICC value of 0.4 (see supplementary Table S2).

The overall within-subject similarity for all contrast conditions exceeded the between-subject similarity values (within: r_self_ = 0.50, r_familiar other_ = 0.19, r_unknown other_ = 0.52, r_self-other_ = 0.23; between: r_self_ = 0.28, r_familiar other_ = 0.04, r_unknown other_ = 0.32, r_self-other_ = 0.09). This translated into a high proportion of participants that could be re-identified based on their neural signature during the different task conditions (i.e., participants can be re-identified if their within-subject correlation coefficients exceeded all between-subject correlation coefficients with other participants). 55% of participants could be re-identified based on their activation during the “self” condition and 69%, when investigating the “unknown other” contrast, while 36% of the sample could be re-identified based on the activation during the “familiar other” condition, and still 30% of participants could be re-identified using the difference contrast (“self-other”).

**Fig. 4:**
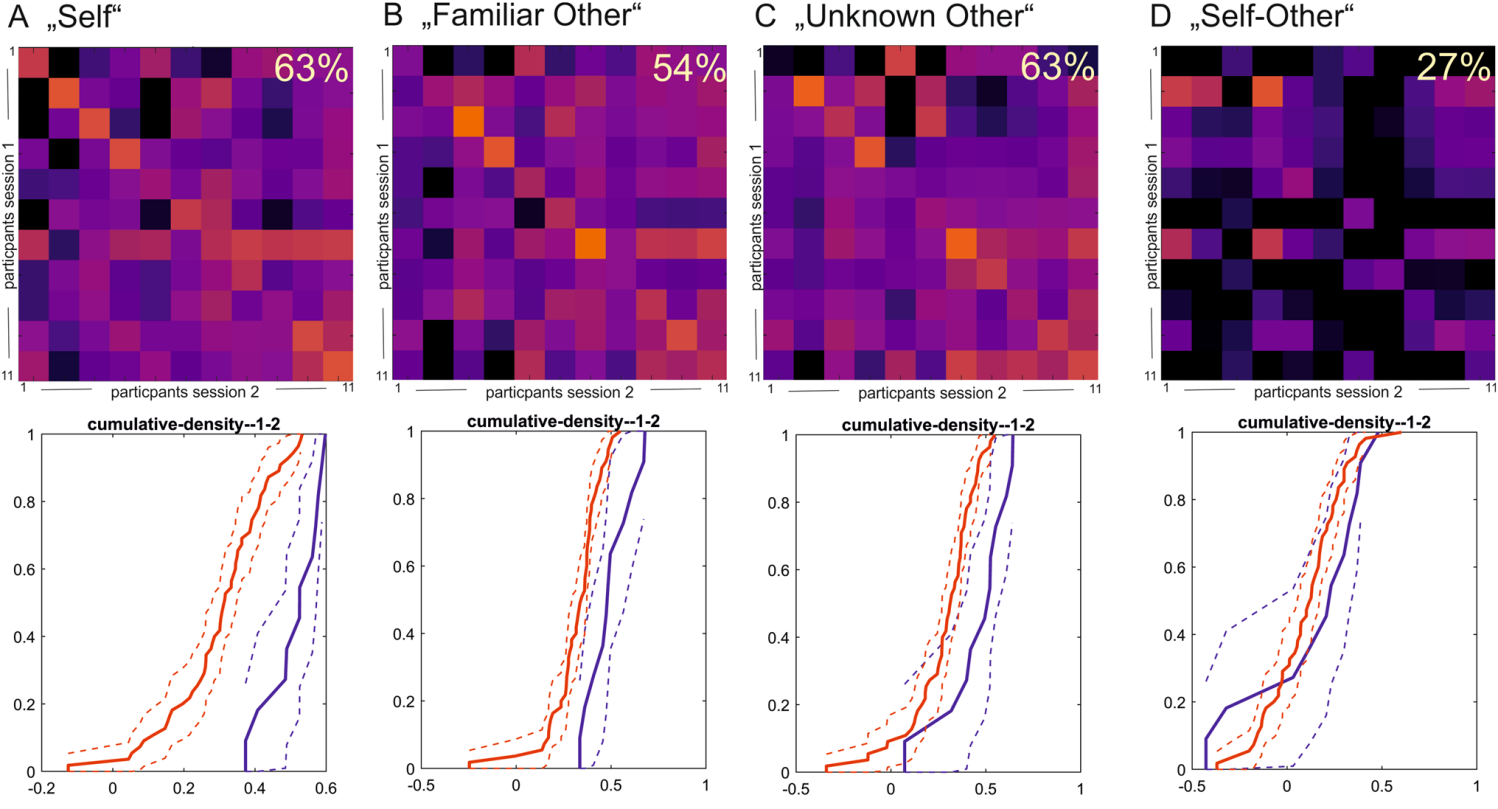
Similarity maps for the patient group (upper row) and empirical cumulative distribution functions (lower row, red lines: between-subject similarity: lower row, blue lines: within-subject similarity) for longitudinal comparisons (first and second fMRI sessions) for the four contrast conditions: (**A**) “self”, (**B**) “familiar person”, (**C**) “unknown person”, and (**D**) “self > familiar and unknown person”. The diagonal of each color matrix represents the within-subject similarity values. Re-identification of a subject based on the neural activation map is affirmed if the within-subject similarity value (diagonal) exceeds all between-subject association coefficients of the same participant (i.e., similarity values in the respective row of the matrix). Higher within-subject similarity is also illustrated by a right-shift of the cumulative density functions for the within-subject similarity values (blue lines) relative to the between-subject similarity values (red lines) for the (**A**) “self”, (**B**) “familiar person”, and (**C**) “unknown person” contrast maps; hereby, the cumulative density functions overlapped for (**D**) the “self > familiar and unknown person” contrast

**Fig. 5:**
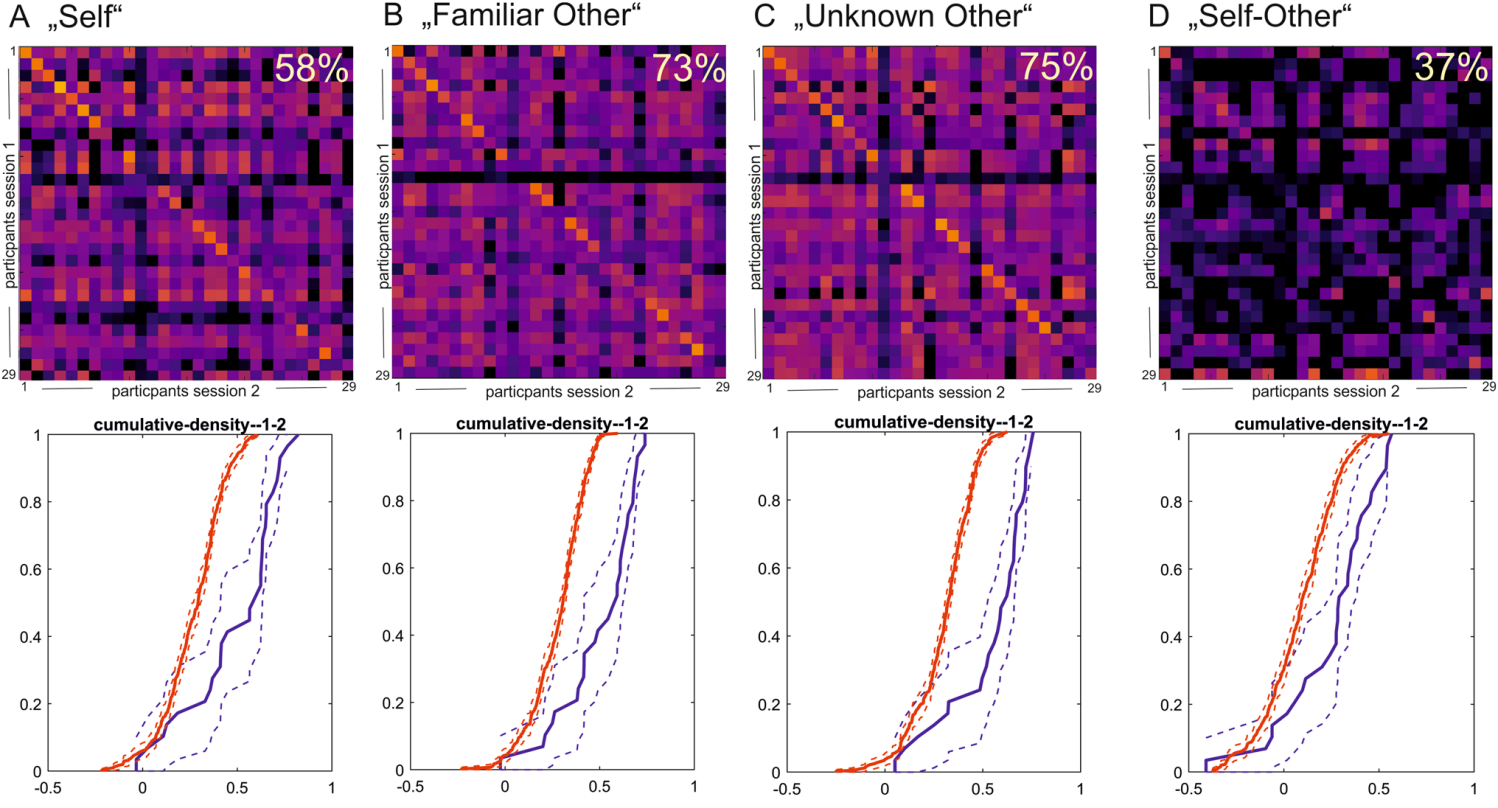
Similarity maps for the control group (upper row) and empirical cumulative distribution functions (lower row, red lines: between-subject similarity; lower row, blue lines: within-subject similarity) for longitudinal comparisons (first and second fMRI sessions) for the four contrast conditions: (**A**) “self”, (**B**) “familiar person”, (**C**) “unknown person”, and (**D**) “self > familiar and unknown person”. The diagonal of each color matrix represents the within-subject similarity values

Results of the similarity analysis demonstrate higher within-subject similarity compared to between-subject similarity from first to second fMRI for the contrast “self > familiar and unknown person” (t_self-familiar+unknown other_ ≥ 5.37, t_self_ ≥ 9.93, *p* < 0.001). In both groups, the overall within-subject similarity exceeded the mean between-subject similarity values (within: r_self-familiar+unknown person r_ ≥ 0.16; between: r_self-familiar+unknown person_ ≤ 0.10). This translated into a proportion of participants that could be re-identified based on their neural signature captured by the contrast “self > familiar and unknown person” of 30% (pooled sample, *N* = 40) with a higher proportion in the group of healthy participants (37%), compared to the groups of individuals with problematic internet use (27%, see Figs. 4 and 5).

The low overall reliability of the “self > familiar and unknown person” contrast also reflected in low Jaccard and Dice coefficients, in the whole study sample and also when analyzing both study groups separately (see Table 4), which indicated that only about 1–8% of significant voxels could be replicated during the second fMRI session. Analyses showed that the magnitude of Jaccard and Dice reliability estimates was significantly lower for the “self > familiar and unknown person” contrast, compared to the three constituent task conditions (F_Jaccard(3,114)_ = 54.386, *p* < 0.001, eta^2^ = 0.589; F_Dice(3,114)_ = 64.886, *p* < 0.001, eta^2^ = 0.631). There was no significant difference between pathological gamers and controls (F_(1,38)Jaccard_ = 0.453, *p* = 0.505, eta^2^ = 0.012; F_(1,38)Dice_ = 0.355, *p* = 0.555, eta^2^ = 0.009).

**Table 4:** Comparison of Jaccard and Dice coefficients across the different task conditions for the pooled sample and both study groups separately

			Contrasts					
			Self	Familiar person	Unknown person	Self > familiar + unknown person	Statistics	*p*
Pooled group (*N* = 40)	Jaccard coefficient	Mean	0.24	0.30	0.32	0.04	F = 78.766	< 0.001*
		(SD)	(0.16)	(0.14)	(0.15)	(0.09)		
	Dice coefficient	Mean	0.36	0.44	0.47	0.07	F = 89.051	< 0.001*
		(SD)	(0.22)	(0.18)	(0.20)	(0.14)		
Controls (*N* = 29)	Jaccard coefficient	Mean	0.25	0.31	0.34	0.05	F = 41.825	< 0.001*
		(SD)	(0.16)	(0.14)	(0.15)	(0.09)		
	Dice coefficient	Mean	0.38	0.45	0.49	0.08	F = 48.060	< 0.001*
		(SD)	(0.21)	(0.19)	(0.19)	(0.14)		
Patients (*N* = 11)	Jaccard coefficient	Mean	0.37	0.43	0.47	0.02	F = 29.128	< 0.001*
		(SD)	(0.23)	(0.12)	(0.15)	(0.03)		
	Dice coefficient	Mean	0.24	0.28	0.32	0.01	F = 34.137	< 0.001*
		(SD)	(0.17)	(0.09)	(0.12)	(0.02)		

Post hoc tests demonstrated a significant difference between the “self > familiar and unknown person” contrast and all other contrast conditions (all *p*’s < 0.003), while the (i) self, (ii) familiar person, and (iii) unknown person contrast conditions did not differ in the magnitude of the Dice and Jaccard coefficients (all *p*’s > 0.05), ﻿* = *p* < 0.05

#### Reliability of the constituent task conditions “self”, “familiar person”, and “unknown person”

The mean ICC values for the (i) self, (ii) familiar person, and (iii) unknown person contrasts were remarkably higher, compared to the contrast “self > familiar and unknown person” in the pooled sample and also when analyzing both study groups separately (see Table 3). Still, the mean ICC values for the pooled dataset (*N* = 40) were below the threshold for moderate reliability (0.4). Thresholded ICC maps for the (i) self, (ii) familiar person, and (iii) unknown person contrast demonstrated good to excellent reliability (1.0 > ICC > 0.75, see Fig. 2) in several brain areas. In the patient group these areas included parts of the bilateral superior; middle and inferior temporal gyrus, including parts of the TPJ; the left and right fusiform gyrus; the right angular gyrus; the middle and inferior occipital gyrus; precuneus; cuneus; superior frontal gyrus; postcentral and precentral gyrus; amygdala; pallidum; insula; cerebellum and parts of the left lingual gyrus; the fusiform gyrus; middle and inferior occipital gyrus; superior, middle, and inferior frontal gyrus; precentral and postcentral gyrus; cuneus; calcarine; and caudate (see supplementary Figure S2). In the control group these areas included parts of the right middle and inferior occipital gyrus; the superior and middle temporal gyrus; cuneus; lingual gyrus; calcarine; precuneus and the left superior; middle and inferior temporal gyrus; the postcentral gyrus; precentral gyrus; Heschl gyrus; supramarginal gyrus; insula; the superior and inferior frontal gyrus and superior occipital gyrus (see supplementary Figure S3).

The atlas-based summary of mean ICC values for the contrast conditions (i) self, (ii) familiar person, and (iii) unknown person showed that several brain regions surpassed the threshold of fair to moderate reliability (ICC > 0.4). These included the bilateral superior; middle and inferior occipital gyri; the superior and middle temporal gyri; the Heschl gyri; the supramarginal gyri; as well as the cuneus, calcarine and lingual gyri (see Supplementary Table S2).

Results of the three constituting contrast conditions show higher within-subject similarity than between-subject similarity from the first to the second fMRI. The difference between within-subject and between-subject similarity is depicted as the prominent diagonal in the similarity matrices (see Figs. 4 and 5). In both groups, the overall within-subject similarity for all contrast conditions exceeded the between-subject similarity values (within: r_self_ >  = 0.50, r_familiar person_ = 0.50, r_unknown person_ = 0.47; between: r_self_ <  = 0.29, r_familiar person_ <  = 0.31, r_unknown person_ <  = 0.33). Analyses based on the pooled dataset (*N* = 40) indicated that 55% of participants could be re-identified based on their activation during the “self” contrast, 69% could be re-identified based on their activation during the “unknown person” contrast and 36% could be re-identified based on their activation during the “familiar person” contrast. Considering the two experimental groups separately, these results showed comparable results. 63% of the participants in the patient group and 58% of the participants in the control group could be re-identified based on their activation during the “self” contrast condition. 54% and 72%, respectively, could be re-identified based on the “unknown person” contrast. 63% and 75%, respectively, could be re-identified based on the activation during the “familiar person” condition (see Figs. 4 and 5).

Analyses of the Jaccard and Dice coefficients for the pooled dataset and considering both experimental groups separately demonstrated a significant main effect in the contrast category for both coefficients, while there was no significant difference between groups. The “self”, “familiar person”, and “unknown person” contrast conditions displayed significantly higher Dice and Jaccard coefficients compared to the “self > familiar and unknown person” contrast (all p’s ≤ 0.003, see Table 4). About 24–47% of the significant voxels for “self”, “familiar person”, and “unknown person” contrasts were replicated during the second fMRI session.

#### Assessment of factors underlying the reliability differences across the contrast conditions

To assess whether the comparatively low reliability of the contrast (“self > familiar and unknown person”) might result from an inter-correlation between the constituting single task condition contrasts, we computed the voxel-wise Pearson correlation coefficient for all three constituting conditions. Data indicate substantial correlations between the three conditions (r_self x familiar person_ = 0.36, SD = 0.26, *R*^2^ = 0.13, r_self x unknown person_ = 0.28, SD = 0.32, *R*^2^ = 0.08, r_unknown person x familiar person_ = 0.29, SD = 0.35, *R*^2^ = 0.08). This evinces that the three constituting task conditions share about 8–12% of their variance. Previous studies indicate that part of the shared variance is removed by subtracting the constituting contrast conditions. In our case, this is illustrated by the lower correlation coefficients between the “self vs. familiar and unknown person” contrast and the constituting contrast conditions (r_(self-familiar+unknown person) x self_ = 0.22, SD = 0.30, *R*^2^ = 0.05; r_(self-familiar+unknown person) x familiar person_ = − 0.01, SD = 0.18, *R*^2^ < 0.01; r_(self-familiar+unknown person) x unknown person_ = − 0.16, SD = 0.24, *R*^2^ = 0.03).

## Discussion

Our study assessed whole-brain longitudinal reliability of an fMRI task that was designed to investigate the neurobiological correlates of participants’ self-concept.

First, we assessed the robustness of group-level brain activation for the task contrast of interest (“self vs. familiar and unknown person”). Results indicate stable brain activation in the bilateral insula; the anterior and medial cingulate; as well as the IFG in both groups at T1. This is in line with the meta-analytical findings of Hu et al. (2016) who report that the ACC, the insula, the IFG as well as regions of the TPJ are activated during reflections and recognition of the own person as compared to other individuals. Furthermore, analyses of psychometric measures that assessed aspects of the self-concept indicated that the vast majority of measures did not show a significant change over time, with moderate to high test–retest reliability of the measures. This supports the notion that self-concept-related aspects can be regarded as relatively stable, thus, confirming the stability of the self-concept construct [25, 29].

Reliability analyses for the contrast of interest “self > familiar and unknown person” showed poor overall reliability, indicated by low mean ICC values and low Jaccard and Dice coefficients. Still, local ICC values indicated fair to moderate reliability in parts of the bilateral middle occipital gyri; the middle and superior temporal gyri; and parts of the TPJ. Similarity analysis indicated that a quarter to a third of the participants could be re-identified based on their neural activation pattern encoded by the contrast “self > familiar and unknown person”. This value might seem unexpectedly high considering the low ICC, Dice, and Jaccard coefficients. However, the very low between-subject correlation values for the contrast in combination with the moderate within-subject correlation coefficients resulted in a re-identification of a substantial proportion of the sample (i.e., the within-subject correlation is higher than all between-subject correlation coefficients). To determine whether the results depended on the sample size, we conducted reliability analyses with the pooled datasets of *n* = 40 participants, in addition to the analyses for the two experimental groups separately. Results of the pooled dataset (*N* = 40) confirm the findings derived from the separate group analyses.

In opposition to the low overall reliability of the “self > familiar and unknown person” contrast, the single constituting contrast conditions “self”, “familiar person”, and “unknown person” showed good to excellent longitudinal reliability in several brain regions associated with the processing of self-referential information [9, 10]. Specifically, this included the bilateral superior and middle temporal and occipital gyri; portions of the TPJ; as well as parts of the cuneus, lingual gyrus, calcarine and areas of the mesolimbic system, such as the insula. The Dice and Jaccard coefficients indicated that about a quarter to a third of the clusters displaying significant activation for the “self”, “familiar person”, and “unknown person” contrasts during the baseline fMRI assessment could be replicated in the second fMRI assessment after 1 year. Similarity analyses also showed that more than half of the participants could be re-identified based on neural activation patterns.

The lower reliability of the contrast “self > familiar and unknown person” might result—at least in part—from a substantial correlation between the constituting contrast conditions. These conditions share about a tenth of their variance, as indicated by the Pearson correlation coefficients. This proportion of shared variance is removed by subtracting the conditions from one another, while the error variances are added. In their recent publication, Infantolino et al. (2018) confirmed that the correlation between the constituting contrast conditions of a contrast, places an upper limit on the reliability of a difference measure. Summarizing the results of 56 independent fMRI studies, a recent meta-analysis showed that only half of the reliability scores fell within the range of at least moderate reliability [4]. The authors concluded that difference scores will always have lower reliability than their constituent contrast conditions; hence, limiting the reliability of such a measure [4].

The sub-perfect reliability of the constituting task condition contrasts points out that additional factors underly the observed limited reliability. Elliot et al. (2020) investigated factors that might limit reliability of task fMRI. Their moderator analysis indicated that neither task type (i.e., process under investigation); task design (e.g., block vs. event-related); task length; test–retest interval; ROI type (i.e., structural vs. functional); nor sample type (i.e., healthy vs. clinical) significantly moderated reliability scores. In the presented analyses, we could not determine the individual factors underlying the sub-perfect reliability of the constituting task condition contrasts. However, it is likely that the self-concept—as well as the associated cognitive processes that are captured by the task under investigation—vary over the test–retest interval of 1 year, limiting the overall reliability. Still, we observed good local reliability of the constituent task conditions, which suggests that fMRI-based measures of the presented task can provide sufficiently reliable estimates of brain activation. In regard to the poor reliability of the contrast “self > familiar and unknown person”, several steps can be undertaken to mitigate the problems associated with computing the contrast between the constituent task conditions. In the case of a linear association and correlation between the constituent task conditions, one task condition can substitute the other condition without losing information on the individual differences between participants [11]. Regarding the self-evaluation task, we argue that the individual response trajectories to pictures of oneself are of special interest. We assume that changes in altered self-concept translate into changes in brain activation captured by the “self” contrast. It can be argued that the focus on a solitary task condition reduces the capacity to isolate specific cognitive processes. This could be overcome by either relying on meta-analysis that could inform on the role of a certain brain region for the cognitive process under investigation. Alternatively, the investigation of within-subject effects between the constituting task conditions could be used to identify regions that specifically activate differently to various task conditions and regions of interest. In a next step, brain activation during the constituting condition “self” could be used as a measure to index individual differences over time with moderate to good or even excellent reliability.

### Limitations

The small sample size of the group of participants with problematic internet gaming use and IGD should be considered a relevant limitation. In addition, the meta-analysis by Elliott et al. (2020) reported that the sample size of studies assessing fMRI reliability ranged from 5 to 58 subjects with a median below 30. While the sample size of the current study should be regarded as a potential limitation, the sample size does, in fact, exceed most previous fMRI reliability studies. Still, the presented findings should be regarded as preliminary and future studies with larger sample sizes are needed.

### Conclusion

To the best of our knowledge, this is the first study that presents longitudinal reliability analyses of a self-evaluation fMRI paradigm. Self-concept-associated brain activation, indexed by the contrast “self > familiar and unknown person” showed poor overall reliability. Still, local reliability measures demonstrated good reliability in regions of the TPJ. Furthermore, similarity analyses indicated that about a third of the participants could be re-identified based on their neural activation, captured by the contrast “self > familiar and unknown person”. In contrast, the reliability estimates consistently indicated good to excellent reliability of the constituting task conditions “self”, “unknown person”, and “familiar person” in several brain regions associated with social cognition. The poor global reliability of the contrast “self > familiar and unknown person” could be explained—at least in part—with a substantial correlation between the brain activation of the constituting contrast conditions. Future fMRI research on self-evaluation should be cautioned by these findings and employ methods to overcome these limitations.

## Supplementary Information

Below is the link to the electronic supplementary material.

Supplementary file1 (DOCX 8288 KB)
