# Supplementary material for: Reliability of the fMRI-based assessment of self-evaluation in individuals with internet gaming disorder
Source: Eur Arch Psychiatry Clin Neurosci. 2021 Jul 17;272(6):1119–34. doi: 10.1007/s00406-021-01307-2 (PMC9388403; doi:10.1007/s00406-021-01307-2)

## Supplementary Tables and Figures

Manuscript: ***Reliability of the fMRI-based assessment of self-evaluation in individuals with internet gaming disorder***

**Supplementary Tables**

**Supplementary Table S1.** Brain areas depicting significantly higher activating during viewing videos of oneself compared to videos of other persons (contrast: “self > familiar + unknown person”, whole-brain threshold p<.001, pFWE, Cluster < .05).

|  |  |  |  |  | ***MNI coordinates*** | | |  |  |
| --- | --- | --- | --- | --- | --- | --- | --- | --- | --- |
| ***H*** | ***Lobe*** | ***BA*** | ***Brain regions*** | ***Cluster size*** | ***X*** | ***Y*** | ***Z*** | ***T*** |  |
| **Pathological gamers (N=11) T1** | | | | | | | | |  |
| L | Frontal | 13/44 | Insula, inferior frontal Gyrus, Gyrus Precentralis | 577 | -40 | 8 | 4 | 5.89 |  |
| R/L | Limbic/Frontal | 32/24 | Anterior and medial Cingulum | 530 | -2 | 38 | 8 | 4.75 |  |
| R | Frontal | 13/47 | Insula, inferior frontal Operculum, Putamen, inferior Frontal Gyrus | 611 | 36 | 16 | 10 | 4.45 |  |
| **Controls (N=29) T1** | | | | | | | | |  |
| L | Frontal/ Temporal | 13/22 | Insula, inferior frontal Gyrus, inferior  frontal Triangularis,  superior temporal gyrus, Claustrum,  Gyrus precentralis | 1149 | -42 | 12 | -2 | 6.66 |  |
| R | Frontal | 45/47 | Insula, inferior frontal Gyrus, inferior  frontal Operculum, Gyrus Precentralis,  Putamen, Claustrum | 834 | 30 | 24 | 6 | 6.26 |  |
| L/R | Limbic/ Frontal | 32/24 | Anterior and medial Cingulum | 1129 | 4 | 28 | 26 | 4.93 |  |
| **All (N=40) T1** | | | | | | | | |  |
| L | Frontal | 13/22/38/44/45/47 | Insula, inferior frontal Gyrus Precentralis, Putamen, superior temporal Gyrus, Claustrum, inferior frontal Operculum, | 1651 | -40 | 10 | 2 | 8.1 |  |
| R | Frontal/temporal | 13/22/44/45/47 | Insula, inferior frontal Gyrus, inferior frontal Operculum, Putamen, Gyrus Precentralis, Globus Pallidum, Superior Temporal Gyrus, Claustrum | 1764 | 32 | 20 | 8 | 6.43 |  |
| L/R | Limbic/Frontal | 24/9/32 | Anterior and medial Cingulum (r and l), superior and medial frontal Gyrus (r), superior Motor Area (r and l) | 1807 | 2 | 28 | 26 | 6.36 |  |
| L/R | Frontal | 6/8 | Superior and medial frontal Gyrus (r), superior Motor Area (r and l) | 585 | -4 | 6 | 62 | 5.99 |  |
| R | Parietal | 1/2/3/40 | Gyrus supramarginalis, Gyrus postcentralis, inferior parietal Lobe, Gyrus Precentralis | 345 | 56 | -30 | 38 | 5.35 |  |
|  | | | | | | | | | |
| **Pathological gamers (N=11) T2** | | | | | | | | | |
| n.s. | | | | | | | | | |
| **Controls (N=29) T2** | | | | | | | | | |
| n.s. | | | | | | | | | |
| **All (N=40) T2** | | | | | | | | | |
| n.s. | | | | | | | | | |
| **Pathological gamers (N=11) T2>T1 and T2<T1** | | | | | | | | | |
| n.s. | | | | | | | | | |
| **Controls (N=29) T2>T1 and T2<T1** | | | | | | | | | |
| n.s. | | | | | | | | | |
| **All (N=40) T2>T1 and T2<T1** | | | | | | | | | |
| n.s. | | | | | | | | | |

*Note.* H = hemisphere; L = left; R = right; BA = Brodmann area; MNI = Montreal Neurological Institute n.s. = not significant; Whole-Brain Threshold p<.001, p*_FWE, Cluster_* < .05).

**Supplementary Table S2.** Atlas-based mean Intraclass Correlation (ICC) values for the four task contrasts “self”, “familiar other”, unknown other” and “self – other” for 120 anatomical regions specified in the aal atlas for the pooled analyses of the whole sample (N=40).

|  | | Contrasts | | | | |
| --- | --- | --- | --- | --- | --- | --- |
| **Brain Region** | **Self** | | **Familiar Person** | **Unknown Person** | **Self > Familiar + Unkown Person** |  |
| 2001_Precentral_L | 0.27 | | 0.38 | 0.37 | 0.03 |  |
| 2002_Precentral_R | 0.37 | | 0.35 | 0.33 | 0.00 |  |
| 2101_Frontal_Sup_2_L | 0.22 | | 0.28 | 0.23 | 0.05 |  |
| 2102_Frontal_Sup_2_R | 0.25 | | 0.29 | 0.28 | -0.01 |  |
| 2201_Frontal_Mid_2_L | 0.17 | | 0.32 | 0.21 | -0.01 |  |
| 2202_Frontal_Mid_2_R | 0.20 | | 0.29 | 0.26 | -0.02 |  |
| 2301_Frontal_Inf_Oper_L | 0.23 | | 0.39 | 0.28 | 0.09 |  |
| 2302_Frontal_Inf_Oper_R | 0.19 | | 0.27 | 0.21 | -0.03 |  |
| 2311_Frontal_Inf_Tri_L | 0.34 | | 0.37 | 0.23 | 0.09 |  |
| 2312_Frontal_Inf_Tri_R | 0.22 | | 0.32 | 0.14 | 0.00 |  |
| 2321_Frontal_Inf_Orb_2_L | 0.21 | | 0.28 | 0.17 | 0.05 |  |
| 2322_Frontal_Inf_Orb_2_R | 0.08 | | 0.29 | 0.17 | -0.06 |  |
| 2331_Rolandic_Oper_L | 0.40 | | **0.53** | **0.40** | 0.09 |  |
| 2332_Rolandic_Oper_R | 0.37 | | **0.46** | 0.29 | 0.03 |  |
| 2401_Supp_Motor_Area_L | 0.36 | | 0.35 | 0.30 | 0.01 |  |
| 2402_Supp_Motor_Area_R | 0.32 | | 0.37 | 0.29 | -0.01 |  |
| 2501_Olfactory_L | 0.23 | | 0.19 | 0.23 | -0.06 |  |
| 2502_Olfactory_R | 0.23 | | 0.19 | 0.21 | -0.13 |  |
| 2601_Frontal_Sup_Medial_L | 0.21 | | 0.30 | 0.24 | 0.04 |  |
| 2602_Frontal_Sup_Medial_R | 0.16 | | 0.26 | 0.25 | -0.01 |  |
| 2611_Frontal_Med_Orb_L | 0.07 | | 0.22 | 0.09 | 0.00 |  |
| 2612_Frontal_Med_Orb_R | 0.03 | | 0.14 | 0.06 | -0.06 |  |
| 2701_Rectus_L | -0.04 | | 0.26 | 0.11 | -0.07 |  |
| 2702_Rectus_R | 0.02 | | 0.18 | 0.07 | -0.18 |  |
| 2801_OFCmed_L | -0.01 | | 0.24 | 0.07 | -0.15 |  |
| 2802_OFCmed_R | 0.04 | | 0.21 | 0.09 | -0.06 |  |
| 2811_OFCant_L | 0.00 | | 0.25 | 0.13 | -0.07 |  |
| 2812_OFCant_R | -0.01 | | 0.20 | 0.09 | -0.02 |  |
| 2821_OFCpost_L | 0.02 | | 0.21 | 0.06 | -0.10 |  |
| 2822_OFCpost_R | 0.07 | | 0.27 | 0.15 | -0.09 |  |
| 2831_OFClat_L | 0.03 | | 0.32 | 0.12 | -0.02 |  |
| 2832_OFClat_R | 0.08 | | 0.26 | 0.02 | 0.02 |  |
| 3001_Insula_L | 0.10 | | 0.28 | 0.17 | -0.02 |  |
| 3002_Insula_R | 0.12 | | 0.29 | 0.16 | -0.04 |  |
| 4001_Cingulate_Ant_L | 0.08 | | 0.25 | 0.18 | 0.03 |  |
| 4002_Cingulate_Ant_R | -0.02 | | 0.24 | 0.16 | 0.06 |  |
| 4011_Cingulate_Mid_L | 0.15 | | 0.36 | 0.21 | -0.02 |  |
| 4012_Cingulate_Mid_R | 0.18 | | 0.33 | 0.24 | 0.01 |  |
| 4021_Cingulate_Post_L | 0.20 | | 0.36 | 0.19 | 0.17 |  |
| 4022_Cingulate_Post_R | 0.09 | | 0.30 | 0.24 | 0.16 |  |
| 4101_Hippocampus_L | 0.07 | | 0.24 | -0.02 | 0.07 |  |
| 4102_Hippocampus_R | 0.06 | | 0.19 | -0.01 | -0.01 |  |
| 4111_ParaHippocampal_L | 0.10 | | 0.22 | 0.14 | -0.02 |  |
| 4112_ParaHippocampal_R | 0.09 | | 0.23 | 0.11 | -0.06 |  |
| 4201_Amygdala_L | 0.20 | | 0.32 | 0.17 | 0.04 |  |
| 4202_Amygdala_R | 0.16 | | 0.28 | 0.08 | -0.08 |  |
| 5001_Calcarine_L | **0.47** | | **0.57** | **0.56** | 0.18 |  |
| 5002_Calcarine_R | **0.54** | | **0.61** | **0.57** | 0.21 |  |
| 5011_Cuneus_L | **0.48** | | **0.49** | **0.53** | 0.14 |  |
| 5012_Cuneus_R | **0.51** | | **0.48** | **0.53** | 0.11 |  |
| 5021_Lingual_L | **0.42** | | **0.52** | **0.43** | 0.09 |  |
| 5022_Lingual_R | **0.45** | | **0.48** | **0.42** | 0.16 |  |
| 5101_Occipital_Sup_L | **0.53** | | **0.45** | **0.53** | 0.22 |  |
| 5102_Occipital_Sup_R | **0.45** | | **0.44** | **0.47** | 0.19 |  |
| 5201_Occipital_Mid_L | **0.43** | | **0.46** | **0.51** | 0.22 |  |
| 5202_Occipital_Mid_R | **0.43** | | **0.41** | **0.44** | 0.29 |  |
| 5301_Occipital_Inf_L | **0.45** | | **0.48** | **0.46** | 0.27 |  |
| 5302_Occipital_Inf_R | **0.43** | | **0.43** | **0.44** | 0.29 |  |
| 5401_Fusiform_L | 0.26 | | 0.33 | 0.32 | 0.07 |  |
| 5402_Fusiform_R | 0.28 | | 0.33 | 0.33 | 0.07 |  |
| 6001_Postcentral_L | 0.27 | | 0.32 | 0.30 | 0.02 |  |
| 6002_Postcentral_R | 0.32 | | 0.31 | 0.31 | 0.07 |  |
| 6101_Parietal_Sup_L | 0.35 | | 0.34 | 0.37 | 0.04 |  |
| 6102_Parietal_Sup_R | 0.38 | | 0.31 | 0.40 | 0.21 |  |
| 6201_Parietal_Inf_L | 0.26 | | 0.39 | 0.33 | 0.03 |  |
| 6202_Parietal_Inf_R | 0.26 | | **0.41** | **0.43** | 0.13 |  |
| 6211_SupraMarginal_L | **0.43** | | **0.57** | **0.45** | 0.11 |  |
| 6212_SupraMarginal_R | 0.31 | | **0.44** | **0.42** | 0.11 |  |
| 6221_Angular_L | 0.29 | | **0.51** | 0.37 | 0.04 |  |
| 6222_Angular_R | 0.20 | | 0.38 | 0.32 | 0.16 |  |
| 6301_Precuneus_L | 0.30 | | **0.41** | 0.34 | 0.09 |  |
| 6302_Precuneus_R | 0.30 | | 0.35 | 0.33 | 0.09 |  |
| 6401_Paracentral_Lobule_L | 0.33 | | 0.30 | 0.27 | 0.04 |  |
| 6402_Paracentral_Lobule_R | 0.35 | | 0.31 | 0.30 | 0.01 |  |
| 7001_Caudate_L | 0.32 | | 0.31 | 0.31 | 0.05 |  |
| 7002_Caudate_R | 0.26 | | 0.30 | 0.30 | 0.03 |  |
| 7011_Putamen_L | 0.22 | | 0.31 | 0.20 | -0.01 |  |
| 7012_Putamen_R | 0.23 | | 0.29 | 0.19 | -0.07 |  |
| 7021_Pallidum_L | 0.26 | | 0.25 | 0.13 | 0.07 |  |
| 7022_Pallidum_R | 0.20 | | 0.21 | 0.14 | -0.01 |  |
| 7101_Thalamus_L | 0.06 | | 0.28 | 0.13 | -0.06 |  |
| 7102_Thalamus_R | 0.06 | | 0.34 | 0.17 | -0.03 |  |
| 8101_Heschl_L | **0.52** | | **0.60** | 0.39 | 0.22 |  |
| 8102_Heschl_R | **0.52** | | **0.61** | **0.41** | 0.15 |  |
| 8111_Temporal_Sup_L | **0.59** | | **0.64** | **0.54** | 0.20 |  |
| 8112_Temporal_Sup_R | **0.56** | | **0.62** | **0.54** | 0.13 |  |
| 8121_Temporal_Pole_Sup_L | 0.21 | | 0.39 | 0.25 | -0.04 |  |
| 8122_Temporal_Pole_Sup_R | 0.27 | | **0.45** | 0.31 | -0.09 |  |
| 8201_Temporal_Mid_L | **0.43** | | **0.51** | **0.47** | 0.09 |  |
| 8202_Temporal_Mid_R | 0.39 | | **0.49** | **0.46** | 0.07 |  |
| 8211_Temporal_Pole_Mid_L | 0.26 | | **0.41** | 0.21 | -0.02 |  |
| 8212_Temporal_Pole_Mid_R | 0.25 | | 0.31 | 0.28 | -0.03 |  |
| 8301_Temporal_Inf_L | 0.13 | | 0.28 | 0.22 | -0.04 |  |
| 8302_Temporal_Inf_R | 0.21 | | 0.22 | 0.28 | 0.01 |  |
| 9001_Cerebelum_Crus1_L | 0.23 | | 0.29 | 0.19 | -0.03 |  |
| 9002_Cerebelum_Crus1_R | 0.21 | | 0.21 | 0.20 | 0.02 |  |
| 9011_Cerebelum_Crus2_L | 0.14 | | 0.26 | 0.12 | -0.15 |  |
| 9012_Cerebelum_Crus2_R | 0.04 | | 0.14 | 0.16 | 0.00 |  |
| 9021_Cerebelum_3_L | 0.08 | | 0.09 | -0.04 | 0.14 |  |
| 9022_Cerebelum_3_R | 0.07 | | 0.14 | 0.06 | 0.04 |  |
| 9031_Cerebelum_4_5_L | 0.14 | | 0.19 | 0.11 | 0.03 |  |
| 9032_Cerebelum_4_5_R | 0.10 | | 0.18 | 0.16 | 0.00 |  |
| 9041_Cerebelum_6_L | 0.28 | | 0.32 | 0.22 | -0.05 |  |
| 9042_Cerebelum_6_R | 0.28 | | 0.27 | 0.25 | -0.05 |  |
| 9051_Cerebelum_7b_L | 0.19 | | 0.18 | 0.08 | -0.12 |  |
| 9052_Cerebelum_7b_R | 0.20 | | 0.19 | 0.14 | 0.16 |  |
| 9061_Cerebelum_8_L | 0.22 | | 0.17 | 0.07 | 0.10 |  |
| 9062_Cerebelum_8_R | 0.30 | | 0.14 | 0.08 | 0.14 |  |
| 9071_Cerebelum_9_L | 0.27 | | 0.05 | 0.12 | 0.11 |  |
| 9072_Cerebelum_9_R | 0.26 | | 0.05 | 0.09 | 0.10 |  |
| 9081_Cerebelum_10_L | 0.18 | | 0.05 | -0.03 | 0.15 |  |
| 9082_Cerebelum_10_R | 0.27s | | -0.01 | 0.14 | 0.15 |  |
| 9100_Vermis_1_2 | 0.20 | | 0.18 | -0.03 | 0.22 |  |
| 9110_Vermis_3 | 0.06 | | 0.14 | -0.02 | 0.21 |  |
| 9120_Vermis_4_5 | 0.09 | | 0.19 | 0.09 | 0.00 |  |
| 9130_Vermis_6 | 0.08 | | 0.22 | 0.11 | -0.11 |  |
| 9140_Vermis_7 | 0.10 | | 0.24 | 0.06 | -0.12 |  |
| 9150_Vermis_8 | 0.18 | | 0.22 | 0.03 | 0.05 |  |
| 9160_Vermis_9 | 0.15 | | 0.17 | 0.04 | 0.07 |  |
| 9170_Vermis_10 | 0.32 | | 0.22 | 0.12 | 0.22 |  |

## Bold font = Areas in which mean ICC values exceed the threshold for moderate reliability (ICC > 0.40)

## Supplementary Figures

**Supplementary Figure S1.** Depiction of brain areas that show significant activation in patients and healthy participants for the different task contrasts: “Self”, “Familiar Person”, “Unknown Person” and “Self > Familiar + Unknown Person” (One-sample t-test, p_FWE_ < .05 whole-brain corrected).


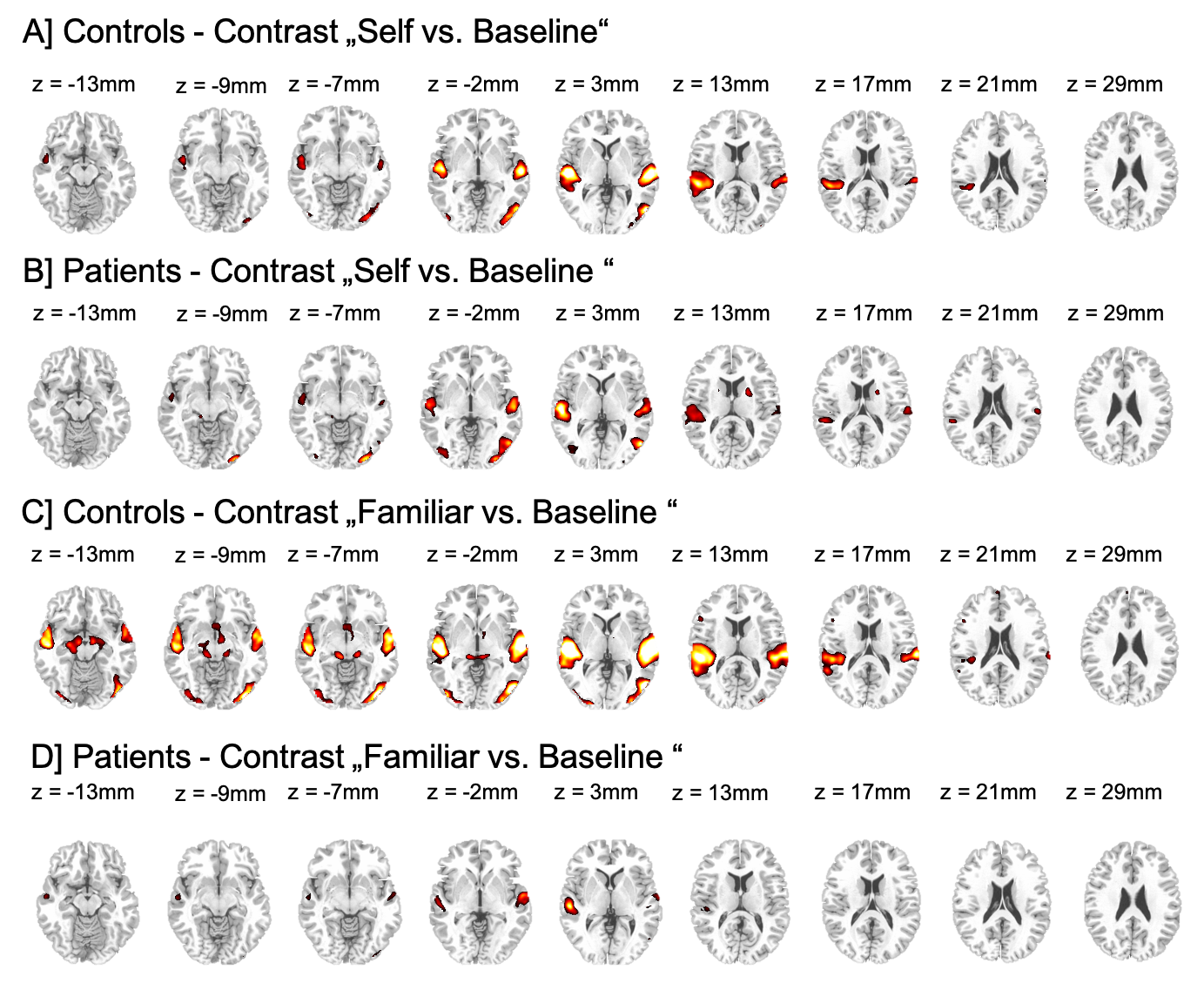


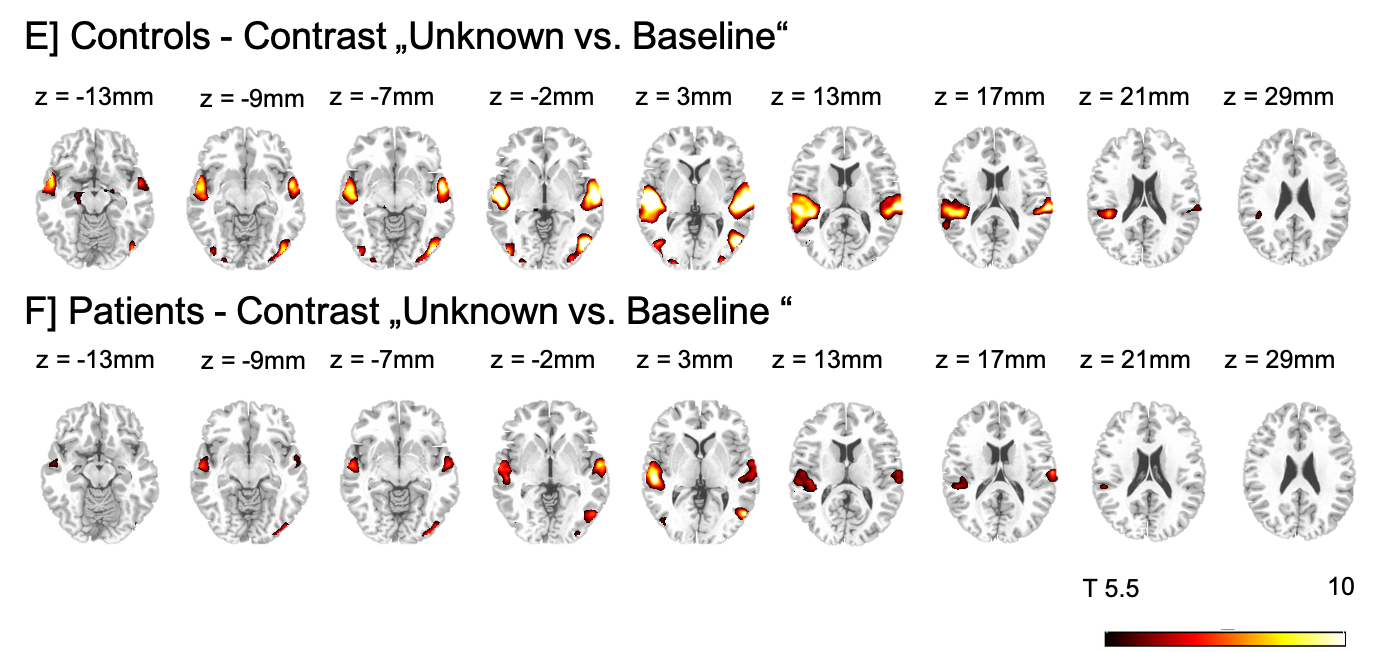


**Supplementary Figure S2.** Depiction of brain areas that show good to excellent reliability (Intraclass correlation [ICC] > 0.75) for the constituent task conditions “self”, “familiar person”, and “unknown person” as well as the contrast “self > familiar and unknown person” in the patient group (N=11).

**
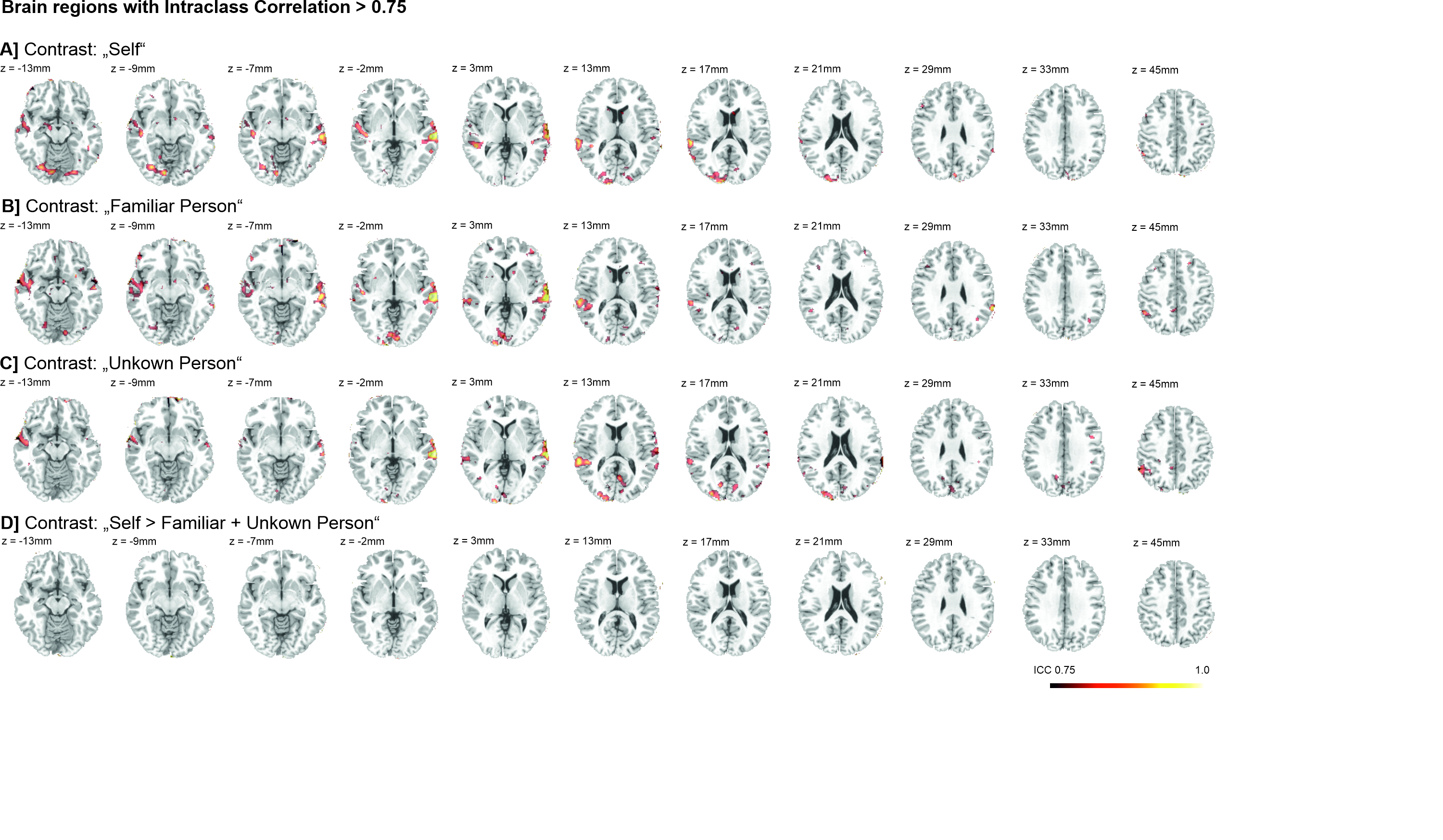
**

**Supplementary Figure S3.** Depiction of brain areas that show good to excellent reliability (Intraclass correlation [ICC] > 0.75) for the constituent task conditions “self”, “familiar person”, and “unknown person” as well as the contrast “self > familiar and unknown person” in the control group (N=29).


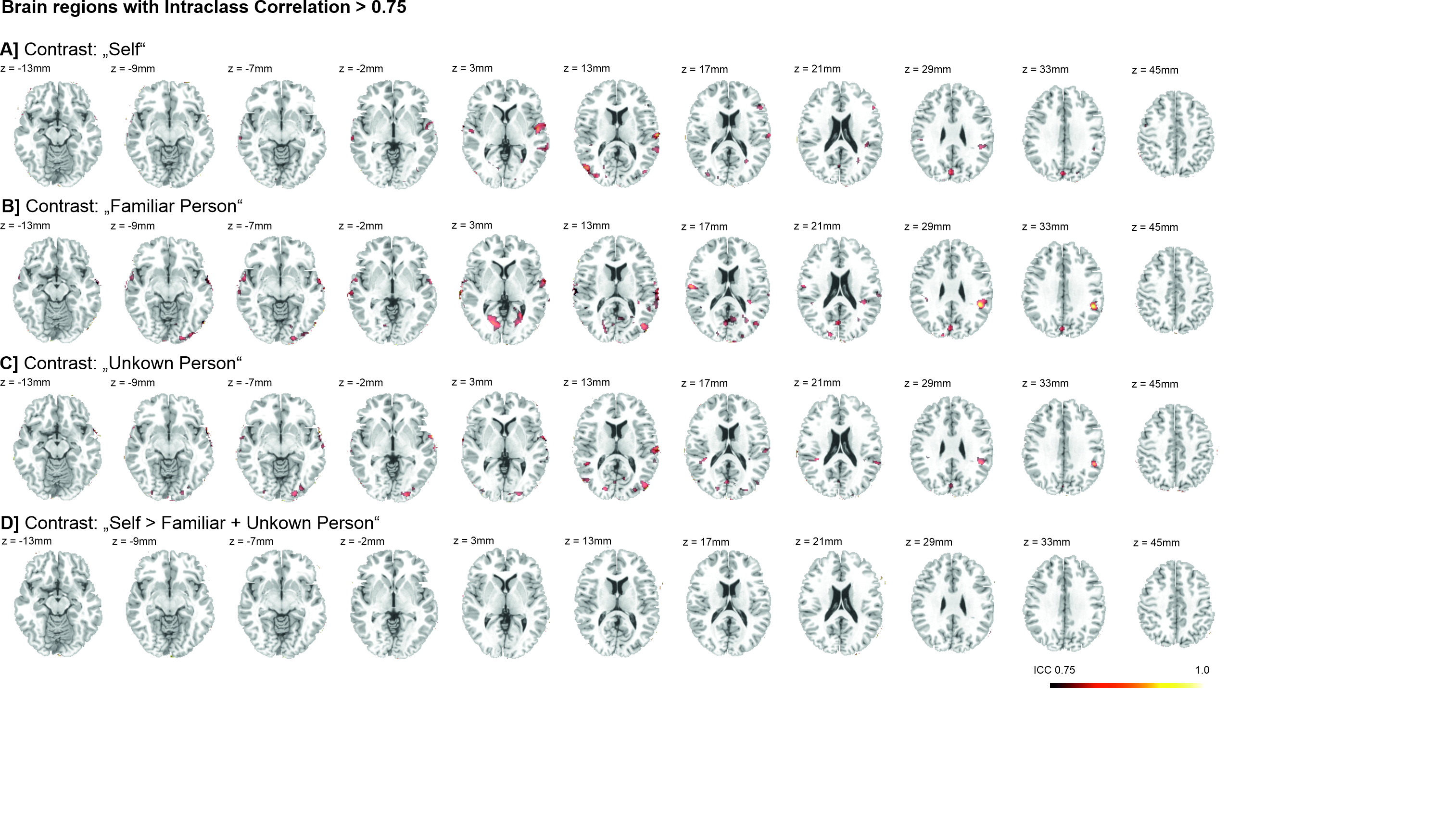

Supplement: Supplementary file 1 — Supplementary file1 (DOCX 8288 KB) [file 406_2021_1307_MOESM1_ESM.docx]
